# A sensitive optical micro-machined ultrasound sensor (OMUS) based on a silicon photonic ring resonator on an acoustical membrane

**DOI:** 10.1038/srep14328

**Published:** 2015-09-22

**Authors:** S.M. Leinders, W.J. Westerveld, J. Pozo, P.L.M.J. van Neer, B. Snyder, P. O’Brien, H.P. Urbach, N. de Jong, M.D. Verweij

**Affiliations:** 1 Lab. of Acoustical Wavefield Imaging, Department of Imaging Physics, Delft University of Technology, P.O.Box 5046, 2600 GA Delft, the Netherlands; 2 Optics Research Group, Department of Imaging Physics, Delft University of Technology, P.O.Box 5046, 2600 GA Delft, the Netherlands; 3 Technical Sciences, TNO, P.O.Box 6000, 2600 JA Delft, the Netherlands; 4 Tyndall National Institute, Photonics Building, Lee Maltings, Cork, Ireland; 5 Department of Biomedical Engineering, Erasmus Medical Center, P.O. Box 2040, 3000 CA Rotterdam, the Netherlands

## Abstract

With the increasing use of ultrasonography, especially in medical imaging, novel fabrication techniques together with novel sensor designs are needed to meet the requirements for future applications like three-dimensional intercardiac and intravascular imaging. These applications require arrays of many small elements to selectively record the sound waves coming from a certain direction. Here we present proof of concept of an optical micro-machined ultrasound sensor (OMUS) fabricated with a semi-industrial CMOS fabrication line. The sensor is based on integrated photonics, which allows for elements with small spatial footprint. We demonstrate that the first prototype is already capable of detecting pressures of 0.4 Pa, which matches the performance of the state of the art piezo-electric transducers while having a 65 times smaller spatial footprint. The sensor is compatible with MRI due to the lack of electronical wiring. Another important benefit of the use of integrated photonics is the easy interrogation of an array of elements. Hence, in future designs only two optical fibers are needed to interrogate an entire array, which minimizes the amount of connections of smart catheters. The demonstrated OMUS has potential applications in medical ultrasound imaging, non destructive testing as well as in flow sensing.

The wide use of ultrasonography, especially in medical imaging, has resulted in the development of different types of ultrasound transducers such as piezo-electric transducers, micro-machined ultrasound transducers (MUTs) and optical ultrasound sensors. Conventional transducers are made of piezo-electric material and nowadays contain multiple piezo elements to form a one or two dimensional array. These arrays enable beam focusing and steering, and hence are able to scan the area or volume of interest and create real-time 2D or 3D images^1^,^2^. The frequencies of the transducers are ranging from 1–20 MHz and typical bandwidths are 70–80%^3^. Recently, Xia *et al.*^4^ presented a piezo-electric array of 5 mm × 5 mm containing 25 elements of 0.9 mm × 0.9 mm. This ultrasound array transducer is used in photoacoustic breast tomography and has a center frequency of 0.9 MHz and a bandwidth of 80%. The noise equivalent pressure is 0.5 Pa per single element. We shall use this element as reference to our sensor.

Because small diagnostic transducers are paramount for extending the possibilities of intravascular and transesophageal ultrasound, one of the goals in the development of piezo-electric transducers is miniaturization^1^. There are two main challenges in the fabrication of small arrays. The first challenge is to obtain the small elements. The conventional fabrication technique uses a diamond saw to divide a single piezo-electric slab into multiple elements^1^. The minimal distance between two neighboring elements is determined by the thickness of the dicing blade (minimal ∼10–15 *μ*m), and advanced fabrication techniques like laser cutting are required when this distance should be decreased.

The second issue is related to the individual wiring of the elements, especially when a large number (order of thousands) of elements is needed^5^,^6^,^7^. Bonding techniques, in which wires are attached to each individual element, are too labor-intensive for arrays containing large amounts of elements. Hence, the use of flexible circuits or other complex techniques are required for the electrical interconnections. Moreover, dense electrical wiring generally brings the disadvantage of intrinsic susceptibility to electromagnetic interference and cross-talk^5^,^7^.

The mentioned production difficulties make that increasing miniaturization of conventional piezo-electric transducer arrays is intricate. To circumvent these difficulties, capacitive and piezo-electric micro-machined ultrasound sensors (CMUTs and PMUTs) are developed^8^,^9^,^10^. A MUT consists of a flexible membrane that is deformable by ultrasonic pressure waves. The micro-machining technology used for the fabrication of MUTs benefits from the developments in the integrated circuit (IC) technology, which allows for a relatively easy fabrication of complex transducer patterns, as well as the integration of accompanying electronic circuitry^11^,^12^. However, these MUTs still have a high electrical impedance resulting in a low signal-to-noise ratio and, consequently, a low imaging depth. To obtain the imaging depth that is currently attained with piezo-electric arrays, the signal-to-noise ratio of MUTs should be improved^3^,^9^.

As far as the receive function is concerned, optical micro-sensors are another alternative for conventional piezo-electric transducers^13^,^14^. These optical devices share the fabrication benefits of MUTs. An additional benefit of these sensors is the lack of electrical wiring or matching circuits. The insensitiveness to electromagnetic interference makes optical devices also compatible with imaging systems such as MRI. In contrast to the previously described electrical transducers, optical devices are not reciprocal and require an external ultrasound source to transmit the sound waves. Ultrasonic optical fiber sensors are commercially available^15^,^16^. However, planar optical circuits are needed to build a dense one or two dimensional array of ultrasound sensors. The group of Guo have reported on sensors that are based on a polymer ring resonator^13^,^17^,^18^. They manufactured a one dimensional array of four polymer micro-ring resonators (100 *μ*m diameter)^18^. Their latest sensor has a noise-equivalent pressure (NEP) of 10.5 Pa for a bandwidth of 1–25 MHz^13^. The fabrication of these polymer photonic integrated circuits is performed with special polymer technology. Rosenthal *et al.*^19^ fabricated optical ultrasound sensors in silicon, using standard silicon IC fabrication technology (CMOS). They reported on a sensor in silicon-on-insulator technology, based on the deformation of a *π*-phase-shift fiber Bragg grating (*π*-FBG) with a length of 250 *μ*m. Currently, the optical modulation in this embedded ultrasound sensor is predominantly caused by the formation of surface acoustic waves. The sensor only has a response to incident pressure waves with an angle larger than 19° to the normal and can therefore not be used for diagnostic ultrasound, which has near normal incidence. Unlike CMUTs and PMUTs, neither of the optical sensors above employ a membrane.

Appreciating the mentioned benefits of both integrated optical sensors and micro-machining technology, we fabricated a receiver-only optical micro-machined ultrasound transducer (OMUS) with a membrane. In doing so we improved the sensitivity by a at least an order of magnitude. Our OMUS contains an optical micro-ring resonator that is integrated onto an acoustical membrane in a silicon chip. We are, to the best of our knowledge, the first to report the operation of such a sensor for ultrasonic frequencies. Photonic integrated circuits in silicon-on-insulator (SOI) technology have a high contrast in refractive index. Therefore, silicon micro-ring resonators can have diameters down to 5 *μ*m without substantial radiation losses^20^. The small spatial footprint of integrated waveguides opens up the possibility to make sensors with sizes in the order of 10 *μ*m, which is an order of magnitude smaller than the current state of the art. Moreover, it allows the addition of small passive optical multiplexers for the simultaneous read-out of many elements^21^,^22^. Hence, the read-out of an entire array of elements can in principle be done with only two optical fibers.

In this paper, we present the measurements of the performance of this novel type of OMUS and show the possibilities of this device as an ultrasound sensor, especially for medical applications.

## Concept, Design and Fabrication

**Figure 1:**
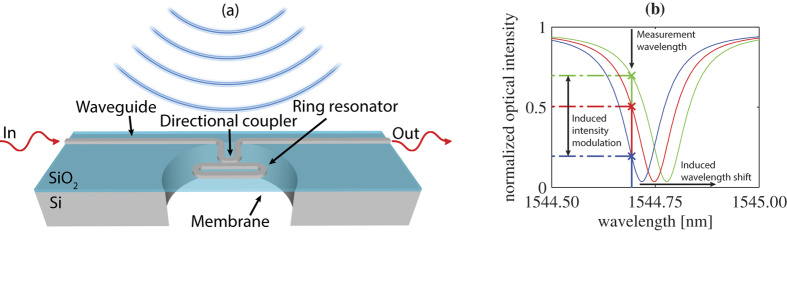
(**a**) Sketch of the OMUS, showing the photonic micro-ring resonator on top of the membrane. (**b**) Sketch of three intensity curves at the end of the waveguide, representing the different transmittances for different strain values of the photonic micro-ring resonator.

The OMUS consists of a waveguide and a photonic ring resonator that are integrated onto a membrane (Fig. 1a). When a broad spectrum of light is transmitted through the waveguide, a part of the spectrum is coupled into the ring resonator by means of a directional coupler^23^. The transmitted spectrum at the output port of the waveguide shows dips at the optical resonance wavelengths *λ*_*m*_ of the ring resonator, given by


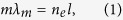


where *m* is an integer number, *l* the circumference of the ring and *n*_*e*_ its effective index of refraction. Incident acoustical pressure waves strain the membrane and hence the resonator. The induced strain in the resonator causes a shift in the optical resonance curves with respect to the undeformed state. The magnitude of this shift due to induced strain depends on three aspects: the amount of elongation of the optical resonator track, the change in effective index of refraction, and the change in dispersion of the material of the track^24^,^25^. The shift in resonance is what we want to observe. To do so without using a dedicated detector, we transmit light, and hence measure the transmitted intensity, at one optical wavelength. This wavelength is chosen on one flank of the optical resonance curve of the undeformed resonator, such that a shift of the resonance curve directly translates into a modulation of the transmitted optical intensity, as is shown in Fig. 1b. The intensity is measured using a photo diode.

The design of the membrane is chosen such that it has a resonance frequency around 1 MHz. To determine the dimensions we use plate bending theory, because the deflection of the membrane is expected to be small compared to its height^26^. With this theory the resonance frequency of the freely moving membrane can be calculated analytically for specific boundary conditions. However, for medical diagnostic purposes the sensor will be submerged in water-like substances, and the predicted resonance frequency in air will shift downwards^27^. Therefore, we use a finite element method (COMSOL Multiphysics) to numerically determine the diameter and thickness of the membrane^28^ that will give the desired resonance frequency. The membrane of the OMUS was designed as a 2.7 *μ*m thick silicon oxide slab with a diameter of 120 *μ*m. It was created by taking silicon with a top layer of silicon dioxide, and etching a hole through the back of the silicon. To ensure a flat bottom surface of the membrane and a reproducible fabrication, all the silicon behind the membrane was etched. The dimensions of the waveguides and directional coupler determine the shape of the optical resonance dips. The shape of the ring resonator and its position on the membrane determine the shift in optical wavelength due to the deformation of the membrane. The steeper the flank of the optical resonances, the more sensitive the sensor. An optimization problem now arises, in that a long racetrack has a narrower resonance dip and therefore a higher sensitivity. However, the strain patterns of a statically deformed membrane showed that a short ring resonator located in the middle of the membrane results in the most strain per pressure. Based on the mechanical FEM analysis and the model in Ref. 23, we chose a racetrack-shaped optical ring-resonator with a bending radius of 5 *μ*m and 40 *μ*m straight track. We use two directional couplers on opposite sides of the ring resonator with a 6% coupling efficiency of optical power. For details on the design, we refer the reader to our previous work^29^,^30^. We use an out-of-plane grating coupler at each end of the waveguide to direct the light upwards out of the waveguides and into optical fibers.

**Figure 2:**
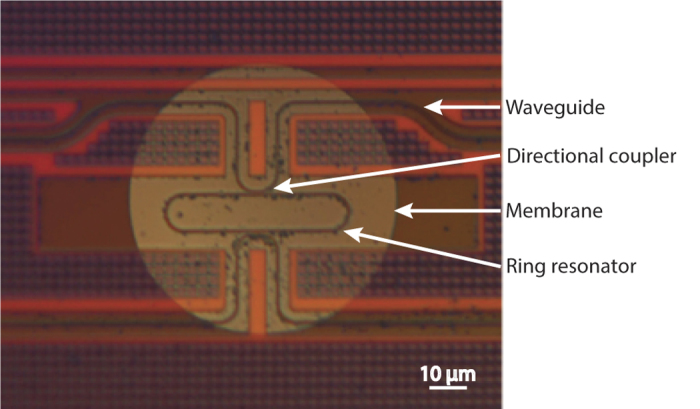
Microscopic image of an OMUS, showing a membrane with an optical resonator and two directional couplers. This OMUS has a circular membrane with a diameter of 78 *μ*m and a thickness of 2.7 *μ*m. The membrane is visible because it is illuminated from the back. The OMUS reported in this paper has a larger membrane diameter of 124 *μ*m.

We realized the sensor using the following procedure. First, the fine optical circuitry was fabricated using a semi-industrial CMOS fabrication line at IMEC (Leuven, Belgium) via the ePIXfab platform^31^. The resulting wafer-piece (die) contains 220 nm high and 400 nm wide silicon waveguides on top of a 2 *μ*m thick silicon-dioxide layer on a 250 *μ*m thick silicon substrate. A 0.5 *μ*m thick silicon-dioxide cladding was deposited to isolate the waveguide from the water. Second, we etched the membrane (124 *μ*m diameter) from the back of the die using deep reactive ion etching with sulfur hexafluoride (SF_6_) as etchant and we used the silicon-dioxide layer as a well-defined etch-stop. A photograph of the membrane with photonic circuitry is shown in Fig. 2. Third, we glued the chips on two 1 mm thick glass plates. The first plate contained a hole of 4 mm diameter, which was positioned behind the membrane. The second glass plate sealed the chip from the backside, ensuring that only air is present behind the membrane. Finally, we connected the optical fibers to the silicon photonic circuit, referred to as packaging. We developed a packaging method that is suitable for under-water usage. This involves attaching to the chip a Pyrex block in which the connecting optical fiber ended. The block had a polished angle facet with an evaporated aluminum coating to reflect light from the fiber into the grating coupler at the end of the waveguide^32^.

## Results

We have optically characterized the OMUS by measuring the transmittance of light. Moreover we performed acoustical measurements to measure the time responses to transmitted acoustical pulses with different frequencies. Finally we determined the bandwidth, sensitivity and noise equivalent pressure of the sensor.

**Figure 3:**
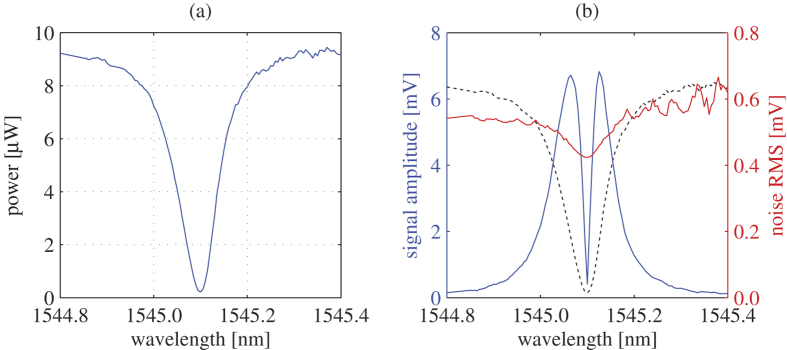
(**a**) The measured transmittance versus optical wavelength, for a maximum output power *P*_0_ of 10 *μ*W. (**b**) The measured output intensity swing of the OMUS (blue line), the RMS value of the noise in the output intensity (red line), and the transmittance (dashed black line; normalized for visibility), all versus the chosen optical wavelength of the laser.

The optical characterization starts by determining the shape of the optical resonance curve of the ring resonator in a static situation, hence without deformation of the membrane. We measured the transmission *T*(*λ*) from the input to the output of the OMUS by stepping through successive optical wavelengths. The intensity *I* at the output of the chip for a static situation is given by *I* = *TI*_0_ with *I*_0_ the maximum output intensity far away from the resonance dip. This is directly related to the maximum output power *P*_0_, which currently is 10 *μ*W (Fig. 3a). The transmittance of the OMUS shows a dip at resonance of the ring resonator which has a FWHM of 100 pm. The transmittance of a membrane at rest is denoted by *T*_0_ and is the initial transmittance of every acoustical measurement. When the membrane and hence the optical resonator is deformed, the transmittance of the ring resonator is shifted. We write the deformed transmittance *T*(*λ*) in first order as *T*(*λ*) ≈ *T*_0_(*λ* + Δ*λ*), where Δ*λ* is the wavelength shift due to the deformation of the ring (see Fig. 1b).

For the acoustical measurements we observe the time modulation of the output intensity at one particular optical wavelength *λ*_*l*_ set by the laser (indicated as measurement wavelength in Fig. 1b). When an incident acoustical wave deforms the membrane and hence the resonator over time, the time dependent intensity at the output of the chip can be described as


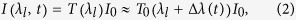


with Δ*λ*(*t*) the ultrasound-induced optical wavelength shift. When we interrogate by measuring the intensity at one particular optical wavelength over time, the sensitivity of interrogation is determined by the gradient of the initial transmittance curve and hence is the largest when the measurement wavelength *λ*_*l*_ is at the point of largest gradient of the transmittance. To show this dependence, we applied an acoustical wave (described in Section Materials and Methods) and measured the maximal output intensity swing versus the chosen measurement wavelength. The result is shown in Fig. 3b (blue line). The curve shows equal sensitivity between the left and right side of the optical resonance dip (dashed black line) as is expected by the symmetry of the resonance dip. The maximal sensitivity is reached at around 1/3 from the bottom of the dip. We also determined the noise versus the chosen optical wavelength (red line) by measuring the intensity fluctuations in the absence of an incident acoustical wave. As is shown in the figure, the noise depends only slightly on the output intensity (dashed black line). Therefore, the signal-to-noise-ratio will mainly depend on the sensitivity curve. For every measurement we will hence use the optical wavelength that gives a maximal output intensity swing; this will be chosen on the left flank.

**Figure 4:**
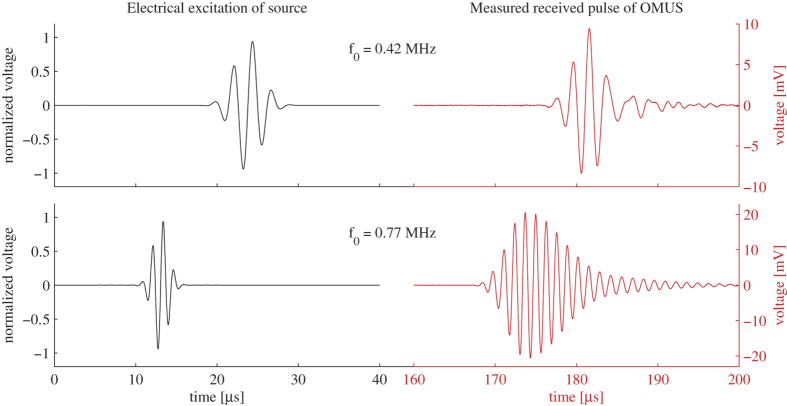
Time responses of the OMUS (right, red) for two transmitted acoustical pulses (left, black) with different center frequencies. The presented signals are an average of 500 individual signals.

After we determined a suitable optical wavelength for our measurements, we investigated the response of the OMUS to an acoustical Gaussian modulated sine wave (see Section Materials and Methods), being transmitted by an external ultrasound source. Two measured time traces of the output signal due to an incident acoustical wave are shown in Fig. 4. The presented signals are an average of 500 individual signals. The upper time trace shows the response to a transmitted acoustical wave with a 0.42 MHz center frequency, which is below the acoustical resonance peak of the membrane. The second time trace shows the response to a transmitted acoustical wave around the resonance frequency of the membrane (*f*_0_ = 0.77 MHz). The signal below the resonance frequency of the membrane resembles the transmitted acoustical pulse quite well, while there is a large acoustical ring-down time present in the signal around the acoustical resonance frequency of the OMUS.

**Figure 5:**
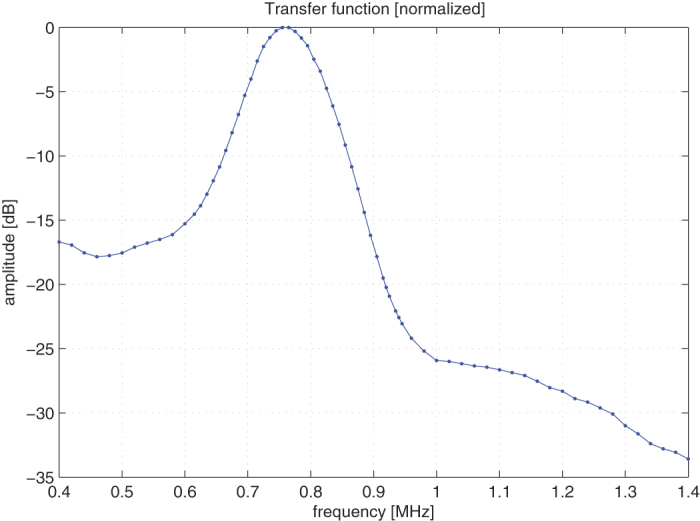
The normalized transfer function of the OMUS.

The normalized transfer function of the OMUS is shown in Fig. 5 and is obtained by sweeping the ultrasound frequency from 0.4 MHz to 1.4 MHz using narrow-band pulses. The −6 dB bandwidth of the OMUS is 19% and the center frequency is 0.76 MHz. The used maximum output power *P*_0_ for these and subsequent measurements was 70 *μ*W.

**Figure 6:**
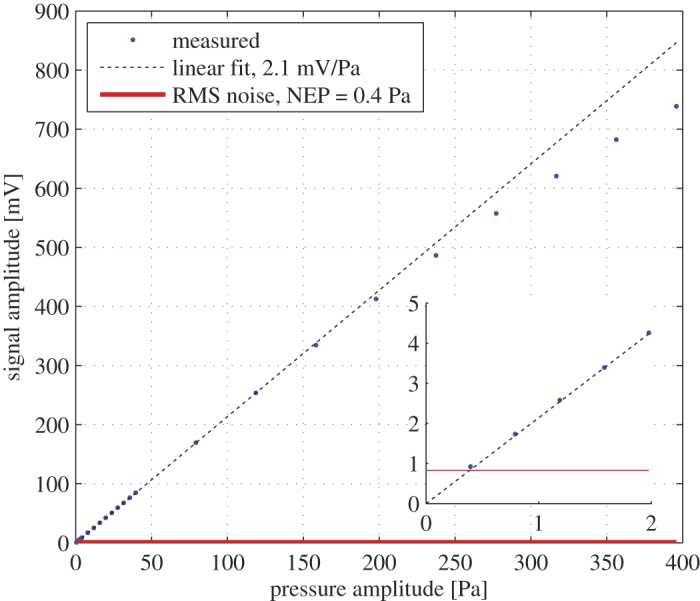
The measured amplitude of the OMUS output signal (blue points) versus the amplitude of the incident pressures, calibrated with a hydrophone. The sensitivity was obtained with a linear fit through the linear region of the system (dashed black line) and has a tangent of 2.1 mV/Pa. The red line is the RMS value of the measured output noise (0.8 mV). The crossing of the lines gives the NEP at 0.4 Pa. The results apply to the center frequency of the OMUS (*f*_0_ = 0.76 MHz).

We determined the sensitivity and noise equivalent pressure of the OMUS for incident acoustic waves with a frequency that corresponds to the maximum of the transfer function (*f*_0_ = 0.76 MHz). The measurement results are shown in Fig. 6 which shows the amplitude of the detected signals versus the amplitude of the exciting acoustical signal. For small deformations of the membrane, and hence for small optical wavelength shifts of the optical resonance, the sensor response is linear. The OMUS has a sensitivity of 2.1 mV/Pa for pressures up till approximately 150 Pa (determined with a linear fit). For higher pressures, the sensitivity curve starts to deviate from a linear behavior. This nonlinear response of the OMUS occurs because of the used detection system. With this system the dynamic range of the OMUS is determined by the width of the optical resonance curve. If the deformation of the membrane, and hence the shift in optical resonance is too large, the response of the OMUS is distorted. We determined a noise equivalent pressure (NEP) of 0.4 Pa by the intersection of the sensitivity curve and the RMS value of the measured output noise (0.8 mV). To transform the NEP value to an equivalent minimum detectable wavelength shift we use the derivative of equation (2) with respect to the wavelength. The minimum detectable wavelength shift Δ*λ*_noise_ is thus calculated from the RMS noise Δ*I*_noise_ as


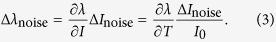


To determine ∂*λ*/∂*T*, we use the derivative of the normalized transmittance (Fig. 3a), resulting in ∂*T*/∂*λ *= 10.5 nm^−1^. With measured values *I*_0_ = 69 *μ*W and Δ*I*_*noise*_ = 21 nW, this results in Δ*λ*_*noise*_ = 29 fm.

## Discussion

The results demonstrate that we fabricated a very sensitive and small ultrasound sensor compared to the polymer ring resonators^13^ and even state of the art piezo-electrical devices. This sensor can easily be repeated into an array of sensors by using waveguide gratings or echelle gratings^21^. These passive optical (de)multiplexers convert a broad spectrum into many wavelength channels and vice versa. When we use grating coupled ring resonators^22^ only one of the many resonance peaks per ring resonator will be present in a wavelength channel, hence allowing to successively stack as many sensors as possible into the available spectrum. Underneath these parallel photonic waveguides with grating coupled ring resonators multiple membranes can be created using dry etching or other techniques^33^. A dense array of sensors is particularly beneficial in the field of intravascular ultrasound, where the outer dimensions of the catheter are restricted to 1 mm and every catheter can only be used once due to hygiene codes. This application will require integration with a separate source (e.g., a piezo-electric element) to form a hybrid transducer. Moreover, an increase of the acoustical center frequency to 15–20 MHz will be necessary, which in principle should be feasible through redimensioning the sensor. Another interesting field of application is photoacoustics where high SNR and a small bandwidth are needed^34^.

Due to its small spatial footprint of 124 *μ*m diameter, the sensor is more prone to noise, which increases with decreasing surface area of the sensor. Nevertheless, the obtained detection limit, or NEP, of 0.4 Pa is better than the NEP of 0.5 Pa offered by the state of the art piezo-electrical device with a 65 times larger surface area.

To optimize our sensor as medical imaging device, there are a few aspects that can be improved. The most important one is addition of absorbing layers or other adjustments to the membrane to increase the bandwidth, which is now 4 times less than for conventional transducers. The resonance frequency of the current OMUS is comparable with the photo-acoustic breast tomography sensor, but needs to be increased for almost all other medical applications. This can be achieved by further reduction of the membrane diameter or by designing a different membrane shape.

Improvements can also be obtained in the dynamic range. With the current detection system, the dynamic range is limited. This could be drastically increased when we use a similar interrogator system as the one used by Rosenthal *et al.*^14^. In their paper, coherence-restored pulse interferometry (CRPI) is introduced which enables them to track the location of an optical resonance peak or dip over time. In that case, the dynamic range will not be limited by the optical interrogation system.

Although further development is needed, with this first prototype we gave proof of the OMUS concept and demonstrated a very sensitive sensor that may form the basis of future ultrasound transducers.

## Method

The optical path of the measurement set-up starts with a tunable laser (Agilent, 81940A) to generate the light. The light passes through an in-fiber isolator (Opto-link Cooration Ltd., OLISO-I-S155), in-fiber attenuator (Opto-link Corporation Ltd., OLVAO-MN-155-2TA) and an in-fiber polarization controller (Thorlabs, FPC560) and is then coupled into the chip. The polarization controller is required because the optical waveguide on the OMUS only accepts one polarization state. The other fiber of the chip is connected to a photo-receiver (Newport, Newfocus 1811-FC-AC).

The acoustical part of the set-up contains a 1 MHz ultrasound transducer (Olympus, Panametrics V314) as source, which is connected to an arbitrary waveform generator (Agilent, 33521A). All the equipment is connected to a computer and we use 16-bit AD-cards (Spectrum, M314142-exp) with a sampling rate up to 250 MSa/s to acquire all the data. The ultrasound source and OMUS are placed in a water basin on a fixed distance of 23.2 cm from each other. The temperature of the water basin is regulated within 0.1 °C by a thermostat (Grant Instruments, GD120) and we use a needle hydrophone (1 mm, Precision Acoustics) to measure the pressure generated at the position of the OMUS.

Every series of measurements starts with a short scan of the optical transmission to determine the optical resonance wavelength. Then we step through the optical wavelengths of the laser, starting from a position halfway the flank, to find the optical wavelength with the highest sensitivity. At this optical wavelength, the successive measurements were performed, by transmitting different sound waves towards the chip. The transmitted acoustical pressure has the shape


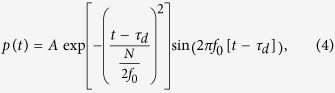


where *p* is the acoustical pressure as a function of time *t*, *A* is the amplitude of the acoustic wave, *τ*_*d*_ is a fixed time delay, *N* is half the number of sine periods that are roughly visible below the gaussian envelope, and *f*_0_ is the center frequency of the sine. We determined the transfer function of the OMUS by transmitting narrow-band pulses (N = 20) at subsequent acoustical frequencies, and adjusted the amplitude in such way that we compensated for the transfer function of the source, as to keep the emitted pressure the same for every acoustical frequency. We measured the sensitivity by altering the amplitude at a fixed frequency of *f*_0_ = 0.76 MHz. The noise measurements were perfomed without a transmitted acoustical signal. We performed every measurement series twice, one with the OMUS and one with the hydrophone, in which the last series provided a reference for the pressure values at the surface of the OMUS.

## Additional Information

**How to cite this article**: Leinders, S.M. *et al.* A sensitive optical micro-machined ultrasound sensor (OMUS) based on a silicon photonic ring resonator on an acoustical membrane. *Sci. Rep.* **5**, 14328; doi: 10.1038/srep14328 (2015).
